# Upregulated Expression of C-X-C Chemokine Receptor 4 Is an Independent Prognostic Predictor for Patients with Gastric Cancer

**DOI:** 10.1371/journal.pone.0071864

**Published:** 2013-08-06

**Authors:** Hongyong He, Cong Wang, Zhenbin Shen, Yong Fang, Xuefei Wang, Weidong Chen, Fenglin Liu, Xinyu Qin, Yihong Sun

**Affiliations:** Department of General Surgery, Zhongshan Hospital, Fudan University, Shanghai, People’s Republic of China; University of Sao Paulo, Brazil

## Abstract

Aberrant chemokine (C-X-C motif) receptor CXCR4 expressions in malignant tissues have been reported, but its role in gastric cancer prognosis remains unknown. Our studies were designed to investigate the expression and prognostic significance of CXCR4 in patients with gastric cancer. CXCR4 expression was retrospectively analyzed by immunohistochemistry in 97 patients with gastric adenocarcinoma from China. Results were assessed for association with clinical features and overall survival by using Kaplan-Meier analysis. Prognostic values of CXCR4 expression and clinical outcomes were evaluated by Cox regression analysis. A molecular prognostic stratification scheme incorporating CXCR4 expression was determined by using receiver operating characteristic (ROC) analysis. The results show that CXCR4 predominantly localized in the cell membranes and cytoplasm. The protein level of CXCR4 was upregulation in gastric cancer tissues and upregulated expression of CXCR4 was only significantly associated with Lauren classification (P<0.001). Increased CXCR4 expression in gastric cancer tissues was positively correlated with poor overall survival of gastric cancer patients (P<0.001). Further multivariate Cox regression analysis suggested that intratumoral CXCR4 expression was an independent prognostic indicator for the disease. Applying the prognostic value of intratumoral CXCR4 density to TNM stage system showed a better prognostic value in patients with gastric cancer. In conclusion, intratumoral CXCR4 expression was recognized as an independent prognostic marker for the overall survival of patients with gastric cancer. On the basis of TNM stage, detection of CXCR4 expression will be helpful for predicting prognosis for patients with gastric cancer.

## Introduction

Despite a marked decline in the incidence of gastric cancer in many industrialized nations, gastric cancer remains the fourth most common neoplasm worldwide and the second most frequent cause of cancer-related mortality partially due to losing curative therapeutic opportunities at the time of initial diagnosis with advanced stage disease in the vast majority of patients [1,2]. Some 400,000 new cases are diagnosed every year in China, accounting for 42% of the world total [3]. Gastric cancer patients with the same stage of the disease present different clinical courses and have different prognosis [4]. This heterogeneity of gastric carcinoma is present at the molecular level and has a genetic predisposition to it [5]. Recently, some molecular-based markers were reported to be significant prognostic factors for patients with gastric cancer [6,7]. However, few of them have been confirmed as independent predictive factor. Therefore, identification of a postoperative useful indicator to better understand the biological basis for the survival of gastric cancer patients may provide important clinically relevant insights into disease management.

Chemokine receptors are expressed not only by leukocytes but also by certain epithelial cells and several types of cancer cell [8]. Previous studies had been demonstrated that CXCL12/CXCR4 axis plays an important role in metastasis of many malignancies, including gastric cancer [9]. CXCL12 is the only known ligand for CXCR4, which activates the receptor CXCR4 and attracts circulating CXCR4-expression cells to peripheral tissues through regulating a wide variety of downstream signal pathways related to proliferation, migration, chemotaxis, and cell survival [10]. It has been demonstrated that cancerous CXCL12 positivity was determined to be an independent prognostic factor for patient survival of gastric cancer [11]. CXCR4 has also been found to be a prognostic marker in various types of cancer, including acute myeloid leukemia [12], breast cancer [13], and prostate cancer [14]. However, a comprehensive analysis of CXCR4 expression in relation to survival of patients with gastric cancer remains largely unknown and needs to be further established.

In the present study, we seek to determine the clinical and prognostic implications of CXCR4 expression in gastric cancer. Our investigation reveals that the protein level of CXCR4 was upregulation in gastric cancer tissues compared with the paired non-tumoral tissues, and its correlation with poor prognosis of patients with gastric cancer was evaluated. Moreover, combination of intratumoral CXCR4 expression and TNM stage showed a better prognostic value than did TNM stage alone.

## Materials and Methods

### Ethics statement

Ethical approval was granted by the Clinical Research Ethics Committee of Zhongshan Hospital of Fudan University (Shanghai, China). Signed informed consent was obtained from all patients for the acquisition and use of patient tissue samples and anonymized clinical data.

### Clinical specimens

We prospectively recruited consecutive patients with gastric cancer, collected the clinicopathologic data and the specimens, and detailed retrospectively analyzed the samples for markers correlating with survival and their role in refining gastric cancer prognostic stratification [15]. Human gastric cancer tissue samples were collected at the time of surgical resection from 97 patients with gastric adenocarcinoma without any chemotherapy or radiation therapy before surgery, which had been formalin-fixed, paraffin-embedded, and clinically and histopathologically diagnosed from January 2000 to December 2005 at Zhongshan Hospital of Fudan University. Non-tumoral gastric tissues were obtained at least 5 cm from the tumor at the same time. Routine chemotherapy had been given to the patients with advanced-stage disease after operation, but no radiation treatment was done in any of patients included in our study. Patients were excluded if they had previously been exposed to any targeted therapy, chemotherapy, radiotherapy, or intervention therapy for gastric cancer. All specimens were pathologically reassessed independently by two gastroenterology pathologists blinded to the clinical data.

### Tissue microarray and immunohistochemistry

Tissue microarrays were constructed as previously described [16]. The tissue microarray paraffin blocks were cut into 4 µm sections. The sections were heated at 70°C for 1 h, dewaxed in xylene and dehydrated through a gradient concentration of alcohol. After retrieving and blocking the endogenous peroxidase and non-specific staining with 3% (v/v) HR_2_ROR_2_R and normal goat serum, the sections were incubated with anti-CXCR4 antibody (R&D systems, Minneapolis, MN, USA) overnight at 4°C. The slides were then incubated with HRP-conjugated goat anti-mouse IgG secondary antibody for 10 min at 37°C. Finally, the sections were visualized by DAB solution (DAKO, Carpinteria, CA, USA) and counterstained with haematoxylin (DAKO, Carpinteria, CA, USA). The specificity of antibody was confirmed by immunohistochemistry with peptide competition. The intensity of immunohistochemistry staining of CXCR4 was scored independently by two gastroenterology pathologists using the semi-quantitative immunoreactivity scoring (IRS) system as previously described [15,17]. Negative controls were treated identically but with the primary antibody omitted.

**Table 1:** Relation between intratumoral CXCR4 expression and clinical characteristics in patients with gastric cancer.

		**Patients**			**CXCR4 Expression**		
**Factor**		No.	%		Low	High	**P**
All patients		97	100		54	43	
Age (years)P^†^							0.266
≤60		48	49.48		24	24	
>60		49	50.52		30	19	
Gender							0.528
Female		35	36.08		18	17	
Male		62	63.92		36	26	
Localization							0.375
Proximal		10	10.31		6	4	
Middle		45	46.39		28	17	
Distal		42	43.30		20	22	
Differentiation							0.160
Well		5	5.16		1	4	
Moderately		36	37.11		23	13	
Poorly		56	57.73		30	26	
Lauren classification							<0.001
Intestinal type		71	73.20		49	22	
Diffuse type		26	26.80		5	21	
T classification							0.767
T1		26	26.80		15	11	
T2		10	10.31		7	3	
T3		4	4.13		2	2	
T4		57	58.76		30	27	
N classification							0.053
N0		38	39.18		22	16	
N1		19	19.59		14	5	
N2		14	14.43		9	5	
N3		26	26.80		9	17	
Distant metastasis							0.697
No		94	96.91		52	42	
Yes		3	3.09		2	1	
TNM stage							0.629
i		30	30.93		19	11	
ii		19	19.59		11	8	
iii		45	46.39		22	23	
iv		3	3.09		2	1	
Tumor size (cm)P^†^							0.450
<3.5		56	57.73		33	23	
≥3.5		41	42.27		21	20	
† Split at median.							

### Statistical analysis

Statistic analysis was performed with MedCalc Software (version 11.4.2.0; MedCalc, Mariakerke, Belgium). Patient baseline characteristics and disease factors were summarized using descriptive statistics. Numerical data were analyzed using Student’s t test, whereas categorical data were studied using Pearson’s χ^2^ For Fisher’s exact test. Cumulative survival time was calculated by Kaplan-Meier method and analyzed by log-rank test. Numbers at risk were calculated for the beginning of each time period. The Cox proportional hazards regression model was used to perform univariate and multivariate analyses. Receiver operating characteristic (ROC) analysis was used to compare the sensitivity and specificity for the prediction of overall survival by the parameters. All P values were two sided, and differences were considered significant at values of P<0.05. Results are reported according to REMARK (Reporting Recommendations for Tumor Marker Prognostic Studies) guidelines [18].

**Figure 1:**
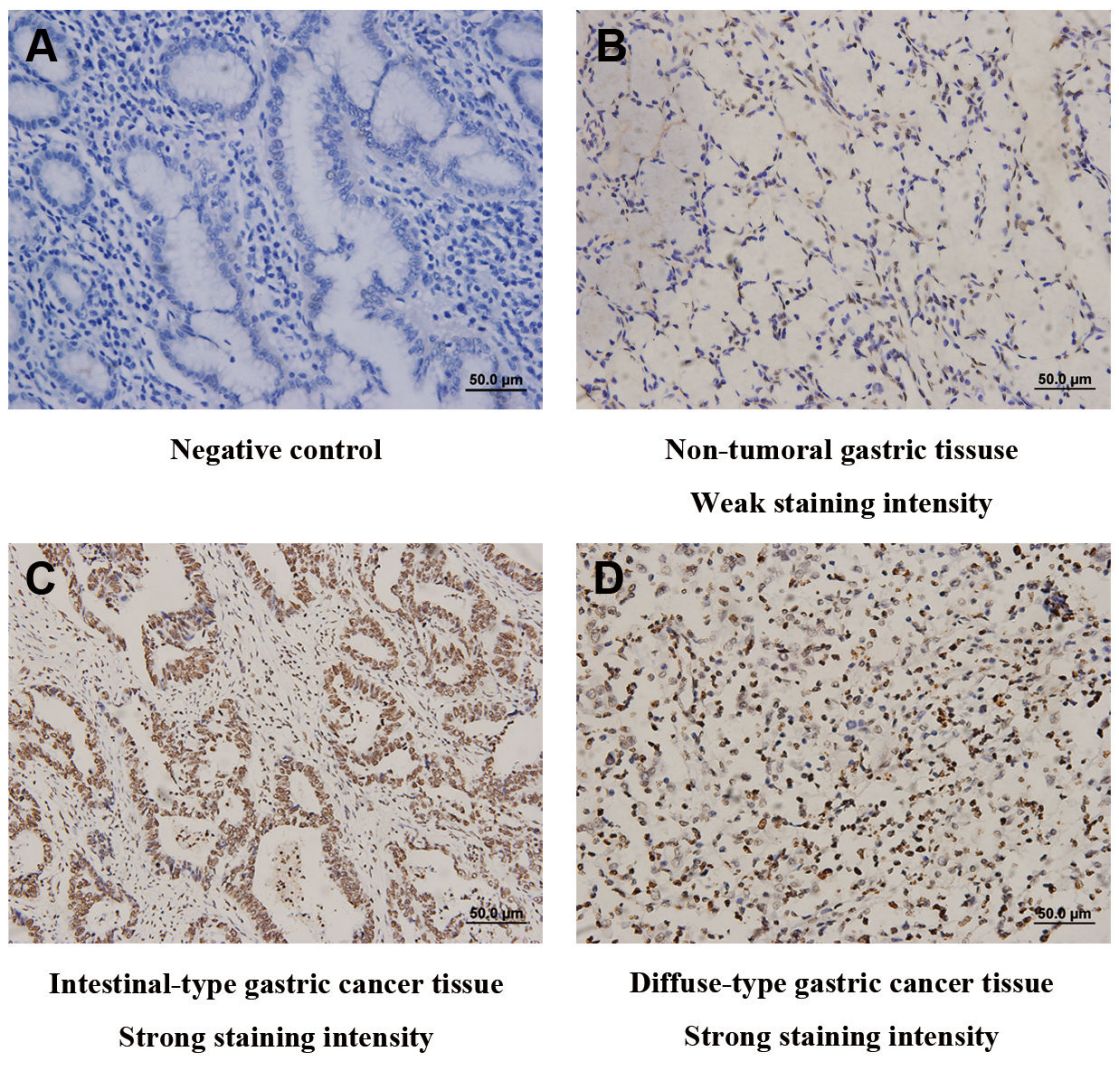
Representative photomicrographs of CXCR4 expression in sections of gastric cancer and non-tumoral tissues. (A) Negative control. (B) Non-tumoral gastric tissue shows week expression of CXCR4. (C) Intestinal-type gastric cancer tissue shows strong expression of CXCR4. (D) Diffuse-type gastric cancer tissue shows strong expression of CXCR4. Scale bar: 50.0 µm.

## Results

### The protein level of CXCR4 was upregulation in gastric cancer tissues

In order to ascertain whether CXCR4 protein is elevated in gastric cancer tissues, we first evaluated CXCR4 expression by immunohistochemical analyses in tumor and paired non-tumoral specimens from 97 patients with gastric cancer. As shown in Figure 1, Most of the stroma cells were negative staining, although sporadic positive staining on these cells was also observed (Figure 1B to 1D). CXCR4 immunoreactivity was mainly in the membranes and cytoplasm of gastric cancer cells (Figure 1C and 1D) and the intensity of the immunohistochemical staining was variable. Compared with week non-tumoral CXCR4 density in gastric epithelial cells (Figure 1B), intratumoral CXCR4 expression increased both in intestinal-type (Figure 1C) and diffuse-type (Figure 1D) gastric cancer. Collectively, these observations suggest that CXCR4 expression is increased in gastric cancer tissues compared with the paired non-tumoral tissues.

### The relationship between CXCR4 expression and clinicopathological factors in patients with gastric cancer

According to the IRS criterion, approximately 44.33% (43 of 97) tumors were scored as high CXCR4 expression. Immunohistochemical staining of CXCR4 levels was statistically analyzed to determine their relationship with various clinicopathologic features of overall 97 gastric cancer patients. As shown in Table 1, intratumoral CXCR4 expression was only associated with Lauren classification (P<0.001). There are more patients with diffuse-type gastric cancer presented high intratumoral CXCR4 expression than with intestinal-type. There are no significant difference in age, gender, primary tumor location, differentiation, T classification, N classification, distant metastasis, TNM stage, or tumor size between the high intratumoral CXCR4 expression group and the low intratumoral CXCR4 expression group. In addition, non-tumoral CXCR4 expression was not associated with any clinicopathlogic factors of gastric cancer patients (data not shown).

### Association of CXCR4 expression with overall survival of patients with gastric cancer

**Figure 2:**
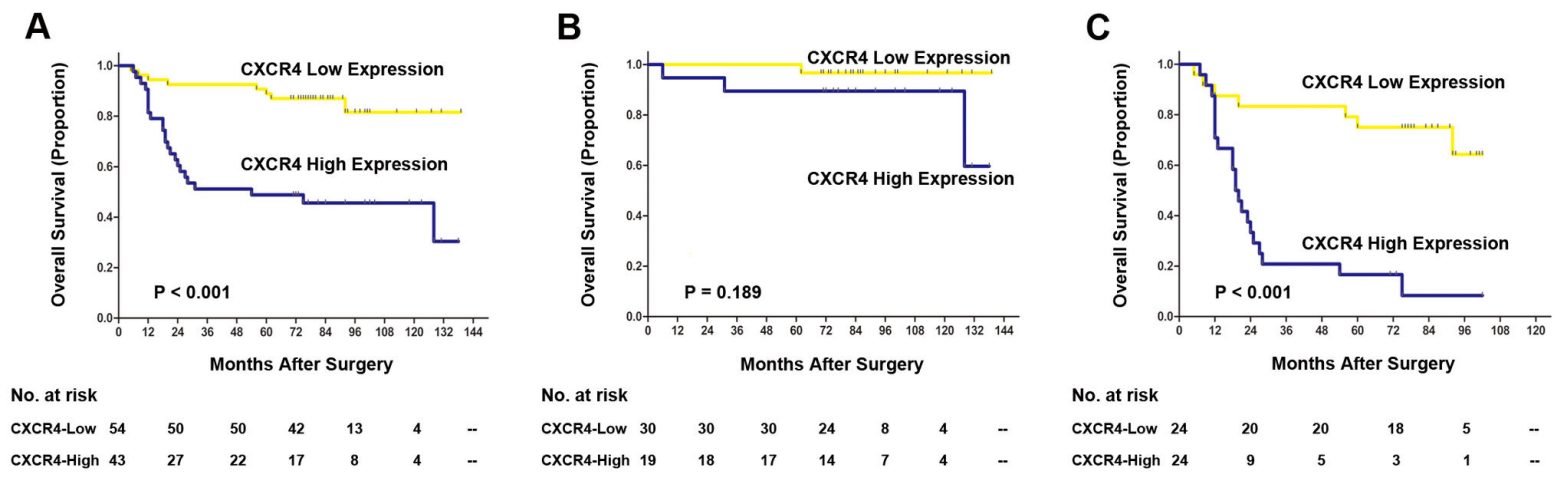
Analyses of overall survival according to the expression of intratumoral CXCR4 in gastric cancer patients. (A–C) Kaplan-Meier analyses of overall survival according to intratumoral CXCR4 expression in patients with gastric cancer in (A) all patients (n=97), (B) TNM stage i+ii (n=49), and (C) TNM stage iii+iv (n=48). P value was calculated by log-rank test.

To further investigate the prognostic value of CXCR4 expression in gastric cancer patients, we compared cancer-specific survival according to intratumoral CXCR4 expression, and Kaplan-Meier survival analysis was performed. As shown in Table 2, Kaplan-Meier survival analysis revealed that the overall survival of gastric cancer patients with high intratumoral CXCR4 expression was significantly poorer than those patients with low CXCR4 intratumoral expression (P<0.001; Figure 2A, Table 2), indicating a crucial impact of intratumoral CXCR4 expression on clinical outcome in patients. However, Kaplan-Meier survival analysis showed that non-tumoral CXCR4 expression was not associated with overall survival of gastric cancer patients (data not shown). In addition, in order to determine whether intratumoral CXCR4 expression could stratify patients with TNM stage stratum, we evaluated the prognostic value of intratumoral CXCR4 expression and did stratified analyses of gastric cancer patients with TNM stage i+ii and TNM stage iii+iv respectively. As shown in Table 2, only the patients with TNM stage iii+iv could be significantly stratified by intratumoral CXCR4 expression, the overall survival time of TNM stage iii+iv patients with low intratumoral CXCR4 expression was significantly longer than those with high intratumoral CXCR4 expression (P<0.001; Figure 2B and 2C, Table 2).

**Table 2:** Log-rank test on overall survival for TNM stage split by CXCR4 expression.

		**Patients**		
**Factor**		No.	%	**P**
All patients		97	100	
CXCR4 expression				<0.001
Low		54	55.67	
High		43	44.33	
TNM stage i+ii		49	50.52	
CXCR4 expression				0.189
Low		30	30.93	
High		19	19.59	
TNM stage iii+iv		48	49.48	
CXCR4 expression				<0.001
Low		24	24.74	
High		24	24.74	

### Upregulated expression of CXCR 4 is an independent prognostic predictor for patient with gastric cancer

In order to estimate the clinical significance of various prognostic factors that might influence survival in the study population, univariate analyses was performed for overall survival in 97 patients with gastric cancer. As shown in Table 3, Lauren classification (P=0.025), T classification (P=0.002), N classification (P=0.001), distant metastasis (P<0.001), TNM stage (P<0.001), and CXCR4 expression (P<0.001) were statistically significant risk factors affecting overall survival of patients with gastric cancer. High intratumoral CXCR4 expression is a significant negative predictor for overall survival (hazard ratio [HR], 4.90; 95% CI, 2.19 to 10.97; P<0.001). To evaluate the robustness of the prognostic value of intratumoral CXCR4 expression, Cox multivariate regression analysis was performed to derive independent risk estimates related to overall survival with the covariates showing significance in univariate analyses. As shown in Table 3, intratumoral CXCR4 expression (HR, 5.07; 95% CI, 2.02 to 12.68; P=0.001) and TNM stage (HR, 15.50; 95% CI, 4.64 to 51.85; P<0.001) were both recognized as independent prognostic factors for overall survival in 97 patients with gastric cancer. Taken together, our findings indicate that intratumoral CXCR4 expression may be a useful marker to predict the survival of patients with gastric cancer.

**Figure 3:**
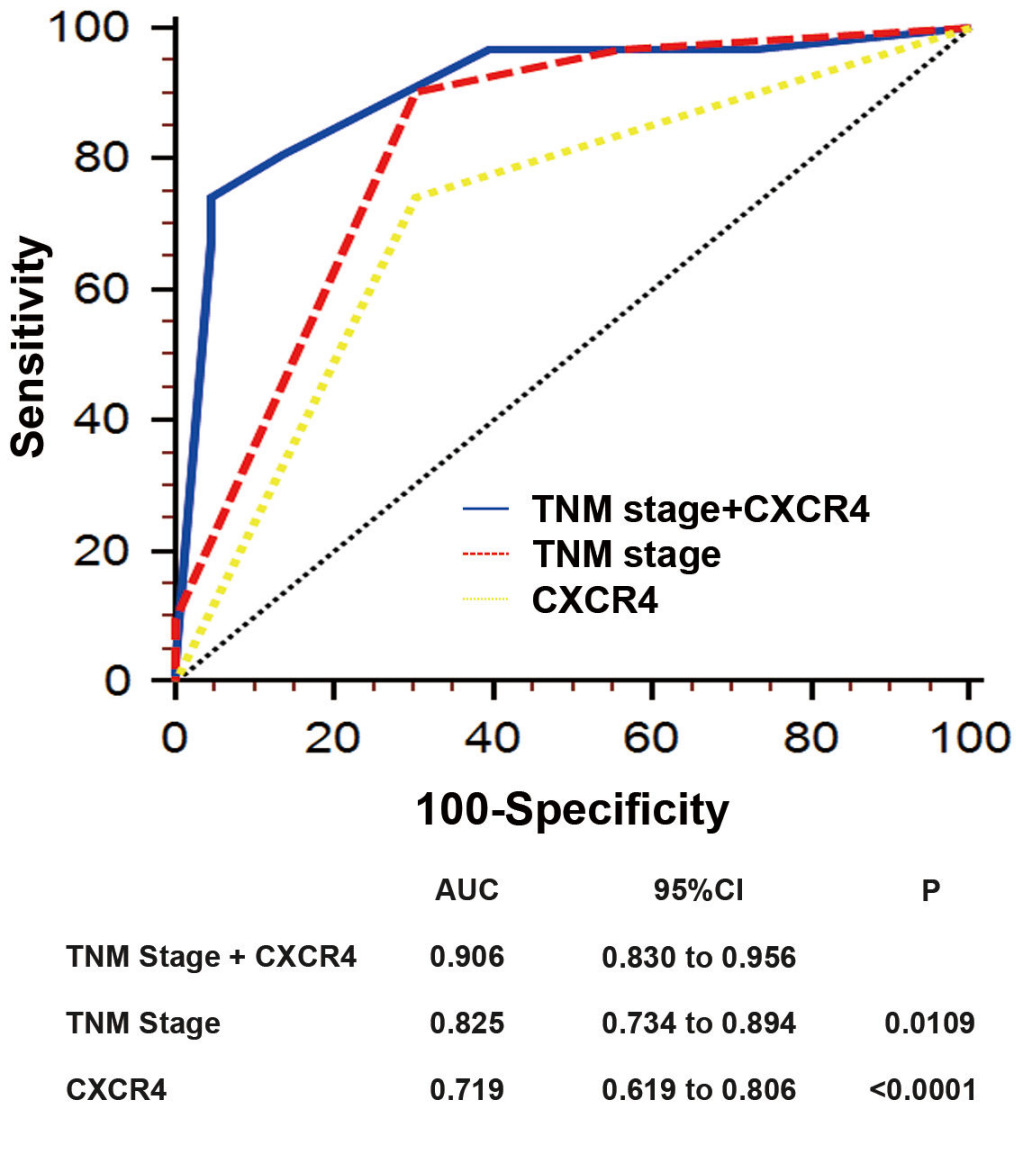
ROC analyses for the prediction of overall survival in patients with gastric cancer. ROC analyses of the sensitivity and specificity for the prediction of overall survival by the combined CXCR4 expression and TNM stage model, the TNM stage model, and the CXCR4 expression model. P values show the area under the ROC curves (AUC) of the combined CXCR4 expression and TNM stage model versus AUCs of the TNM stage model or the CXCR4 expression model. ROC, receiver operating characteristic.

**Table 3:** Univariate and multivariate Cox regression analyses for overall survival in patients with gastric cancer.

		**Overall Survival**	
**Univariate**		**Hazard Radio (95%CI)**	**P**
Age (years)P^†^P: >60 vs ≤60		1.61 (0.79 to 3.27)	0.190
Gender: Male vs Female		0.98 (0.46 to 2.05)	0.948
Localization: Distal vs Proximal+ Middle		1.52 (0.74 to 3.12)	0.251
Differentiation: Poorly vs Well+ Moderately		1.63 (0.76 to 3.50)	0.206
Lauren classification: Diffuse vs Intestinal		2.31 (1.11 to 4.80)	0.025
T classification: T3+T4 vs T1+T2		23.36 (3.18 to 171.71)	0.002
N classification : N1+N2+N3 vs N0		25.79 (3.51 to 189.58)	0.001
Distant metastasis: Yes vs No		14.54 (3.84 to 55.06)	<0.001
TNM stage: iii+IV vs I+ii		13.12 (3.97 to 43.41)	<0.001
Tumor size (cm)P^†^P: ≥3.5 vs <3.5		1.13 (0.55 to 2.33)	0.736
CXCR4 expression: High vs Low		4.90 (2.19 to 10.97)	<0.001
**Multivariate**		**Hazard Radio (95%CI)**	**P**
Lauren classification: Diffuse vs Intestinal		1.29 (0.59 to 2.82)	0.520
TNM stage: iii+IV vs I+ii		15.50 (4.64 to 51.85)	<0.001
CXCR4 expression: High vs Low		5.07 (2.02 to 12.68)	0.001

Abbreviation: 95% CI, 95% confidence interval

† Split at median.

### Extension of the TNM stage prognostic model with intratumoral CXCR4 expression

To develop a more sensitive predictive tool, we constructed a prognostic score model combining two independent prognostic factors, intratumoral CXCR4 expression and TNM stage, and compared its prognostic validity with the intratumoral CXCR4 expression alone and TNM stage alone models by means of receiver operating characteristic (ROC) analysis. Combination of intratumoral CXCR4 expression and TNM stage (AUC [95% CI], 0.906 [0.830 to 0.956]) showed a better prognostic value than did TNM stage (AUC [95% CI], 0.825 [0.734 to 0.894]; P=0.0109) or intratumoral CXCR4 expression (AUC [95% CI], 0.719 [0.619 to 0.806]; P<0.0001) alone (Figure 3).

## Discussion

Advanced gastric cancer prognosis tends to be dismal, despite aggressive therapy [19]. Defining molecular subgroups may identify patients who could benefit from targeted therapies and personalized treatment is regarded as the best option to reduce gastric cancer mortality rates [2,20]. In the development and metastasis to distant organs, gastric cancer shares many similarities with leukocyte trafficking, which is crucially regulated by chemokines and their receptors [21]. The chemokine receptor CXCR4 belongs to the large supperfamily of G protein-coupled receptors and has been identified to play crucial roles in the homing of haematopoietic cells and the metastasis of many solid tumors [22]. In addition, CXCR4 has also been found to be a prognostic marker in various types of cancer, including acute myeloid leukemia [12], breast cancer [13], and prostate cancer [14]. In our study, we first identified high intratumoral CXCR4 expression as an independent poor prognostic factor for overall survival following gastrectomy of gastric cancer patients, and only the patients with TNM stage iii+iv could be significantly stratified by intratumoral CXCR4 expression. Moreover, in this study, incorporation of intratumoral CXCR4 density into current clinicopathologic TNM stage system improved prognostic value for overall survival. These data suggest that the intratumoral CXCR4 density might add some prognostic information for patients with gastric cancer and lead to a more accurate classification under the TNM stage system. However, the results of integration of intratumoral CXCR4 expression into current prognostic model and the potential clinical practice changing should be validated in an independent and larger data set. And the profound molecular roles of CXCR4 in gastric cancer progression remain far from being fully elucidated and await further investigation.

Chemotherapy is an indispensable element of treatment for gastric cancer patients, and fluoropyrimidines, platinum-containing agents, alone or in combinations, are the most effective and commonly used chemotherapy regimens [23]. Metastasis and drug resistance are major problems in gastric cancer chemotherapy [24]. Previous study had been reported that CXCR4 was a potential marker for docetaxel chemosensitivity in gastric cancer [25]. In our study, we demonstrated that the overall survival of gastric cancer patients presented high intratumoral CXCR4 expression with TNM stage iii+iv was significantly poorer than those patients with low CXCR4 intratumoral expression (P<0.001; Figure 2C, Table 2). These gastric cancer patients with TNM stage iii+iv were routinely treated with chemotherapy postoperation. These data suggest that intratumoral CXCR4 expression may be a potential predictive marker in chemotherapy sensitivity of gastric cancer. While the molecular mechanisms underlying the drug-resistance gastric cancer cells need to be analyzed in the future.

It has been reported that metastasis was associated with chemokine signaling through the CXCL12/CXCR4 axis in many tumors, including gastric cancer [26]. Cancerous CXCL12 positivity was determined to be an independent prognostic factor for patient survival of gastric cancer and CXCR4 expression was associated with lymph node and liver metastasis of gastric cancer [9,11]. Kwak et al. investigated the CXCR4 expression immumohistochemically in more than three hundred gastric cancer patients, they concluded that there were no significant clinical implications of CXCR4 expression in gastric cancer except for tumor histology, and the expression of the chemokine receptor CXCR4 was found to be high in differentiated and intestinal-type gastric cancers [27]. However, in our study, there is no significant association between CXCR4 expression and clinical characteristics of gastric cancer except for Lauren classification, and there are more patients with diffuse-type gastric cancer presented high intratumoral CXCR4 expression than with intestinal-type. This contrary finding might reflect the different IRS criterion and different genetic background of patients with gastric cancer, and the profound molecular roles of CXCR4 in gastric cancer differentiation remain far from fully understood and merit further investigation. In addition, CXCR7 is also a receptor for CXCL12 that binds this chemokine with greater affinity [28], the clinical and prognostic implications of CXCL12/CXCR4/CXCR7 axis in patients with gastric cancer remain to be elucidated in future.

CXCR4 signaling has been implicated in tumor growth, intravasation, migration, and survival, and optimal use of CXCR4 inhibition may be a part of potential multimodality therapy for gastric cancer treatment [29]. The bicyclam AMD3100 is known as a small synthetic inhibitor of the CXCL12-binding chemokine receptors CXCR4 and CXCR7 [30]. AMD3100 could disrupt the interaction of tumor cells with the microenvironment of distant metastasis sites and enhance the sensitivity to therapy [31]. And the safety of AMD3100 has been established in clinical trials in which the drug was delivered either as a single dose or as a continuous infusion. Previous study has been demonstrated CXCR4 antagonists maybe useful for the treatment of peritoneal carcinomatosis of gastric cancer in a mouse model [32]. These data suggest that targeting CXCL12/CXCR4 signal with AMD3100 may be a novel and efficient strategy for the treatment in advanced gastric cancer patients with distant metastasis potential.

In summary, our results demonstrate that increased intratumoral CXCR4 expression predicts independently poor postoperative overall survival of patients with gastric cancer. Integration of intratumoral CXCR4 density into current clinicopathologic TNM stage system might add some prognostic information for patients with gastric cancer.
